# Taxonomic status of the Columbia duskysnail (Truncatelloidea, Amnicolidae, *Colligyrus*)

**DOI:** 10.3897/zookeys.514.9919

**Published:** 2015-07-22

**Authors:** Hsiu-Ping Liu, Robert Hershler, Christopher S. Rossel

**Affiliations:** 1 Department of Biology, Metropolitan State University of Denver, Denver, CO 80217, USA; 2 Department of Invertebrate Zoology, Smithsonian Institution, P.O. Box 37012, Washington, DC 20013-7012, USA; 3 United States Department of Agriculture, Forest Service, Mt. Hood National Forest, Barlow Ranger District, 780 NE Court Street, Dufur, OR 97021, USA

**Keywords:** Gastropoda, aquatic, western United States, systematics, phylogeny, conservation

## Abstract

Undescribed freshwater snails (Amnicolidae: *Colligyrus*) from the Mount Hood region (northwestern United States) identified as a new species (commonly known as the Columbia duskysnail) in grey literature have been provided federal protection under the “survey and manage” provisions of the Northwest Forest Plan and have been placed on conservation watch lists. However, there are no published studies of the identity of these snails aside from a molecular phylogenetic analysis which delineated a close relationship between the single sampled population and *Colligyrus greggi*, which is distributed more than 750 km to the east of the Mount Hood area. Here we examine the taxonomic status of the Columbia duskysnail based on additional molecular sampling of mitochondrial DNA sequences (COI) and morphological evidence. We found that the Columbia duskysnail is not a monophyletic group and forms a strongly supported clade with *Colligyrus greggi*. The COI divergence between these broadly disjunct groups (2.1%) was somewhat larger than that within *Colligyrus greggi* (1.0%) but considerably less than that among the three currently recognized species of *Colligyrus* (8.7–12.1%). Additionally we found that the Columbia duskysnail and *Colligyrus greggi* cannot be consistently differentiated by previously reported diagnostic characters (size and shape of shell spire, pigmentation of body and penis) and are closely similar in other aspects of morphology. Based on these results we conclude that the Columbia duskysnail is conspecific with *Colligyrus greggi*.

## Introduction

**Figure 1.**
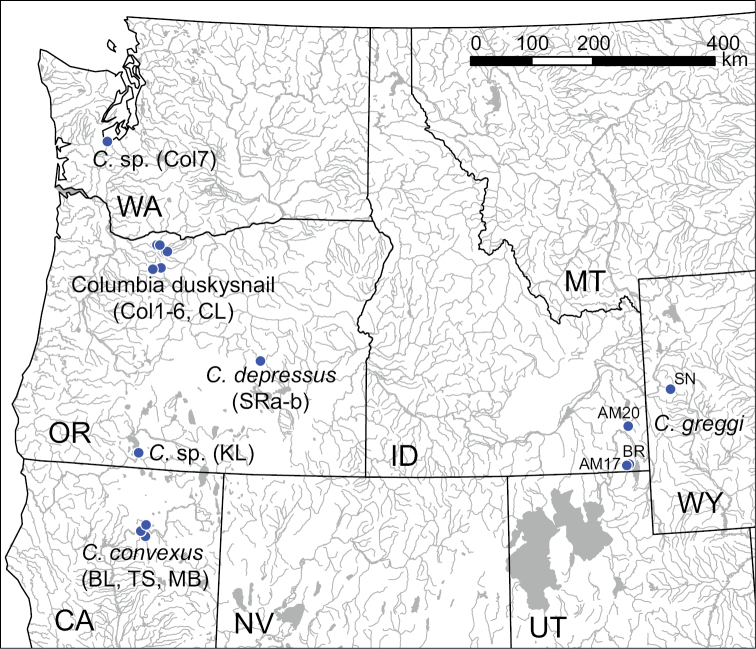
Map of the northwestern United States showing the collecting localities for *Colligyrus* samples used in the molecular analysis. Specimen codes are from Table 1.

The freshwater gastropod genus *Colligyrus* contains three currently recognized species (commonly known as duskysnails) that live in cold water seeps and springs in the northwestern United States (Hershler 1999, Hershler et al. 2003). *Colligyrus greggi* is distributed in the upper Snake River drainage and a small portion of the northeastern Great Basin while the other two congeners are narrowly ranging in the northwest Great Basin (*Colligyrus depressus*) and Pit River drainage (*Colligyrus convexus*) (Fig. 1). There are also numerous undescribed populations in other portions of the northwestern United States (e.g., Klamath River basin) that may belong to this little studied genus.

**Table 1.** Samples used for molecular analysis, with codes (used in Figs 1–2), locality details, and GenBank accession numbers for COI.

Taxon	Code	Locality (voucher catalog number)	GenBank accession number
Columbia duskysnail	COL1 (*N*=4)	Small spring, Brooks Meadow, middle Columbia River basin, Hood River Co., OR	KT248021
	COL2 (*N*=4)	Spring tributary, Tony Creek, middle Columbia River basin, Hood River Co., OR	KT248022
	COL3 (*N*=4)	Bottle Prairie, middle Columbia River basin, Hood River Co., OR	KT248023
	COL4 (*N*=4)	Spring tributary, Ramsey Creek, middle Columbia River basin, Wasco Co., OR	KT248024
	COL5 (*N*=4)	Spring tributary, Clear Creek, Deschutes River basin, Wasco Co., OR	KT248025 (COL5A, *N*=3) KT248026 (COL5C, *N*=1)
	COL6 (*N*=4)	Bear Creek, Hood River Co., OR	KT248027 (COL6A, *N*=3) KT248028 (COL6C, *N*=1)
	CL	Oak Grove Fork, Willamette River basin, Clackamas Co., OR	AY196174
*Colligyrus convexus*	BL	Baum Lake, Pit River basin, Shasta Co., CA	AY196166
	TS	Fall River (spring source), Pit River basin, Shasta Co., CA	AY196167
	MBa	Burney Creek, Pit River basin, Shasta Co., CA	AY196168
	MBb	Burney Creek, Pit River basin, Shasta Co., CA	AY196169
*Colligyrus depressus*	SRa	Second spring south of Turner Ranch, Silvies River basin, Harney Co., OR	AY196170
	SRb	Third spring south of Turner Ranch, Silvies River basin, Harney Co., OR	AY196171
*Colligyrus greggi*	SN	Springs along Cliff Creek, upper Snake River basin, Sublette Co., WY	AY196172
	BR	Spring at Saint Charles campground, Bear Lake basin, Bear Lake Co., ID	AY196173
	AM17 (*N*=2)	Spring at Porcupine campground, Bear Lake basin, Bear Lake Co, ID	KT248030
	AM20 (*N*=2)	Springs along Trail Creek, upper Snake River basin, Caribou Co., ID	KT248031
*Colligyrus* sp.	KL	Link River, Klamath River basin, Klamath Co., OR	AY196175
*Colligyrus*? sp.	COL7 (*N*=1)	Allison Springs, Puget Sound drainage, Thurston Co., WA	KT248029
*Amnicola limosa*	-	Blind Lake, Lake Michigan basin, Washtenow Co., MI	AF213348

The cluster of undescribed duskysnail populations in the vicinity of Mount Hood (Columbia River basin) was identified in grey literature as a new species, commonly known as the Columbia duskysnail (Frest and Johannes 1993), and differentiated from morphologically similar *Colligyrus depressus* by its smaller size; and from *Colligyrus greggi* by its smaller, less attenuated (shell) spire, and lighter pigmentation of the body and penis (Frest and Johannes 1995). The description of this putative novelty did not include supporting data, illustrations, or voucher details. There have been no subsequently published studies of the Columbia duskysnail aside from a molecular phylogenetic analysis of *Colligyrus* (Hershler et al. 2003, fig. 6) which delineated a close relationship between the population in Oak Grove Fork (Willamette River basin) and *Colligyrus greggi*, which is distributed more than 750 km to the east of the Mount Hood area.

The Columbia duskysnail has received considerable attention from the conservation community owing to its supposedly narrow distribution, and threats that include road construction, logging, and water diversions (USFWS 2011). It was listed as a Record of Decision (ROD) Survey and Manage species under the Northwest Forest Plan (USDA and USDI 1994) and has been placed on several conservation watch lists (e.g., NatureServe 2015). However, in response to a recent listing petition, the USFWS (2012) found that addition of the Columbia duskysnail to the federal list of threatened or endangered species was not warranted at this time owing to the absence of published evidence that it is a “listable entity” (i.e., a distinct species).

Clearly there is a need to clarify the taxonomic status of the Columbia duskysnail as a prerequisite for protection under the Endangered Species Act and other possible conservation measures. Here we address this research gap by further analysis of mtCOI sequences (for which six populations of these animals and two populations of *Colligyrus greggi* were newly sampled) and assessment of reported diagnostic morphologic characters.

## Methods

For the molecular component of this study we newly sampled two populations of *Colligyrus greggi*, six populations of the Columbia duskysnail from the Lower Deschutes River and Middle Columbia-Hood River basins, and a population of another putatively undescribed species of duskysnail (from the Puget Sound region) recognized in grey literature (Johannes 2010). Specimens were preserved in 90% ethanol in the field. Genomic DNA was extracted from entire snails (1–4 specimens per sample) using a CTAB protocol (Bucklin 1992); each specimen was analyzed for mtDNA individually. LCO1490 (Folmer et al. 1994) and COH743 (5’GGT AAA ATT AAA ATA TAT ACT T3’) were used to amplify a 720 base pair (bp) fragment of COI. Amplification conditions and sequencing of amplified polymerase chain reaction product followed Liu et al. (2003). Sequences were determined for both strands and then edited and aligned using Sequencher^©^ version 5.0.1. The 29 newly sequenced specimens were analyzed together with our previously published *Colligyrus* dataset (Hershler et al. 2003); *Amnicola limosa* (AF213348) was used as the root in each analysis. One example of each haplotype detected in a given sample was used in the analyses. The new haplotypes from each sampling locality were deposited in GenBank (accession numbers KT248021–KT248031). Sample information and GenBank accession numbers are given in Table 1; the locations of the *Colligyrus* populations from which sequences were obtained are shown in Figure 1.

MrModeltest 2.3 (Nylander 2004) was used to obtain an appropriate substitution model (using the Akaike Information Criterion) and parameter values for the molecular phylogenetic analyses. This program selected HKY + I model parameters as the best fit model for the COI dataset. Phylogenetic analyses were performed using four different methodologies—distance, maximum parsimony (MP), maximum likelihood (ML) and Bayesian inference. The distance, MP, and ML analyses were performed using PAUP*4.ob10 (Swofford 2002), and the Bayesian analyses were conducted using MrBayes 3.2.3 (Ronquist and Huelsenbeck 2003). For the distance analyses, HKY distance was used to generate a neighbor-joining (NJ) tree (Saitou and Nei 1987). The MP analyses were conducted with equal weighting, using the heuristic search option with tree bisection reconnection branch-swapping and 100 random additions. The ML analyses were performed using the HKY + I model; a HKY distance based NJ tree was used as the initial topology for branch-swapping. Node support was evaluated by 10,000 bootstrap pseudo-replicates except for the ML analysis, for which support values were based on 1000 replications. For the Bayesian analyses Metropolis-coupled Markov chain Monte Carlo simulations were run with four chains (using the model selected through MrModeltest) for 2,000,000 generations. Markov chains were sampled at intervals of 10 generations to obtain 200,000 sample points. We used the default settings for the priors on topologies and the HKY + I model parameters selected by MrModeltest as the best fit model for both analyses. At the end of the analyses, the average standard deviation of split frequencies was 0.0018 and the Potential Scale Reduction Factor (PSRF) was 1, indicating that the runs had reached convergence. The sampled trees with branch lengths were used to generate a 50% majority rule consensus tree, with the first 25% of the samples removed to ensure that the chain sampled a stationary portion.

Genetic distances within and between samples were calculated using MEGA6 (Tamura et al. 2013), with standard errors estimated by 1,000 bootstrap replications with pairwise deletion of missing data. Since MEGA does not contain the HKY model that was selected by MRMODELTEST, we used the Tajima-Nei distance, which is the nearest model.

The morphologic component of the study was focused in large part on evaluating the purported diagnostic differences between the Columbia duskysnail and *Colligyrus greggi*. Shell parameters were compiled for two samples of the former and five samples of the latter to assess variation in spire size and shape, and other aspects of shell form. Ten to 20 adult specimens (having fully formed apertural lips) were selected from amongst the largest specimens of each sample. The height of the entire shell (SH), width of the body whorl (WBW), and height of the aperture (AH) were measured from camera lucida outline drawings using a digitizing pad linked to a personal computer (see Hershler 1989). Ratios were generated from the raw data to estimate the size of the spire relative to aperture height (SH-AH/AH) and shape of the spire (SH-AH/WBW). Sample heterogeneity of these parameters was examined through analysis of variance (ANOVA), with post-hoc testing of differences among means using the Bonferroni correction for multiple comparisons. We also performed a discriminant analysis of seven standard shell parameters (total number of whorls, height and width of entire shell, body whorl, and aperture) obtained from this same set of specimens (measurement methods as above). A classification matrix based on the resulting canonical scores was generated to assess accuracy of assignment of individual specimens to the Columbia duskysnail and *Colligyrus greggi*. Analyses were performed using Systat for Windows 11.00.00 (SSI 2004). Several recently collected ethanol-preserved samples of the Columbia duskysnail were examined to assess purportedly diagnostic (soft part) pigmentation patterns, and to further evaluate the distinctiveness of these animals relative to *Colligyrus greggi*. Variation in the number of cusps on the radular teeth (*N*=5) was assessed using the method of Hershler et al. (2007).

## Results

**Figure 2.**
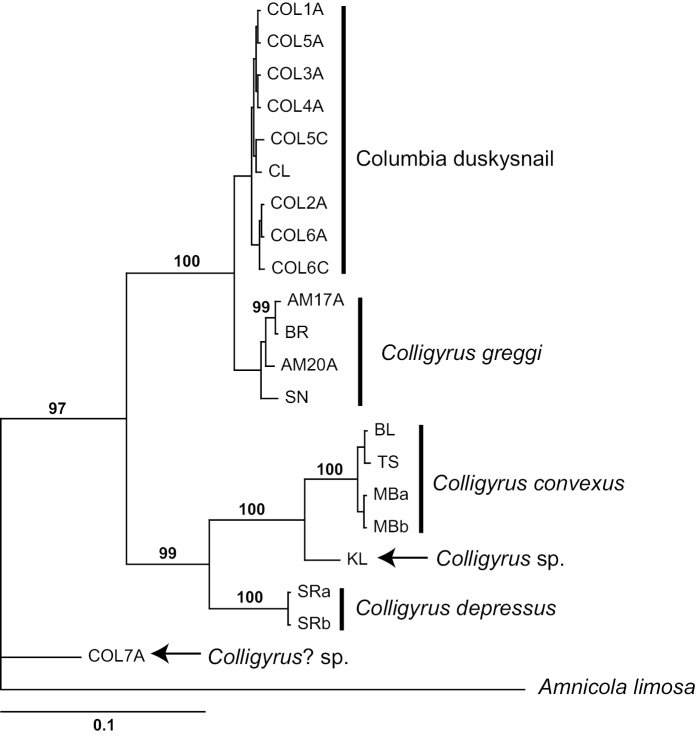
Bayesian tree based on the COI dataset. Nodes having posterior probabilities >95% are shown. Specimen codes are from Table 1.

The Columbia duskysnail COI sequences formed a strongly supported clade with *Colligyrus greggi* in all but the ML tree; the Bayesian topology is shown in Figure 2. This clade differed genetically from *Colligyrus convexus* and *Colligyrus depressus* by >10% (Table 2). The Columbia duskysnail and *Colligyrus greggi* differed from each other by 2.1 ± 0.5% (ranging from 1.7–2.7%) and formed mutually exclusive sub-clades (albeit without strong support) in all but the ML tree in which the latter formed a clade while the former was paraphyletic. There was little variation among Columbia duskysnail specimens (0.3 ± 0.1%, ranging from 0.0–0.8%) and somewhat greater variation within *Colligyrus greggi* (1.0 ± 0.3%, ranging from 0.2–1.5%). Note that the sequenced specimen from the Puget Sound area (Col7A) was positioned basally outside of the *Colligyrus* clade in all of the resulting trees.

**Table 2.** Mean mtCOI sequence divergence (Tajima-Nei distance) among amnicolid lineages. Values are percentage ± standard deviation.

Lineage	*Colligyrus greggi* + Columbia duskysnail	*Colligyrus convexus*	*Colligyrus depressus*	*Colligyrus* sp. (KL)	*Colligyrus* sp. (COL7)
*Colligyrus greggi* + Columbia duskysnail	1.2 ± 0.3				
*Colligyrus convexus*	11.6 ± 1.5	0.0. ± 0.2			
*Colligyrus depressus*	10.5 ± 1.4	8.7 ± 1.3	0.0 ± 0.0		
*Colligyrus* sp. (KL)	10.7 ± 1.4	4.0 ± 0.9	8.5 ± 1.3		
*Colligyrus* sp. (COL7)	12.1 ± 1.5	12.8 ± 1.7	13.2 ± 1.6	11.9 ± 1.5	
*Amnicola limosa*	19.0 ± 1.9	19.2 ± 2.1	19.6 ± 2.1	19.4 ± 2.1	16.4 ± 1.8

**Figure 3.**
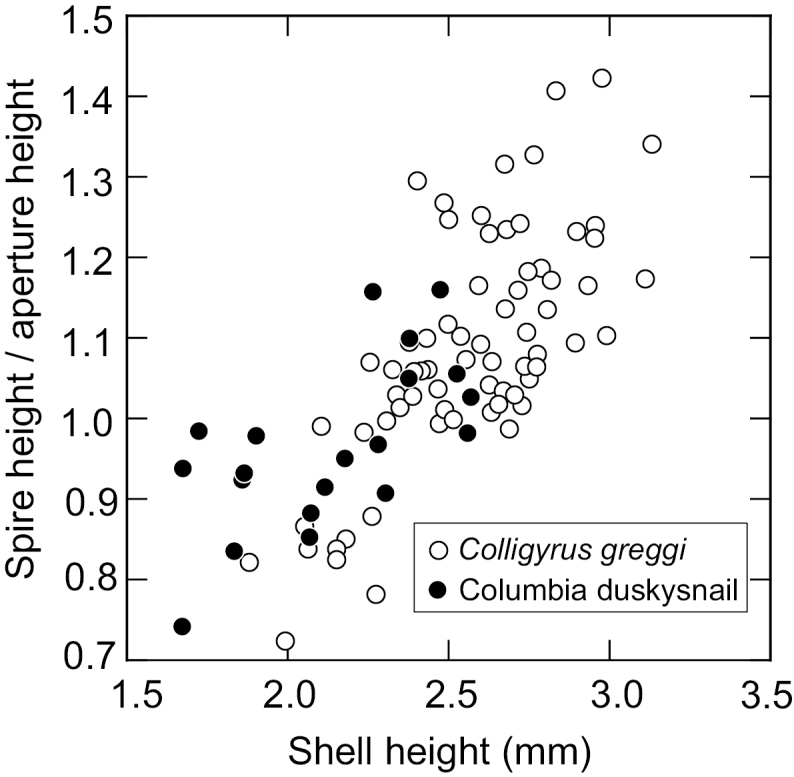
Scatterplot of shell size (SH + SW) and spire size (SH-AH/WBW) for specimens from five samples of *Colligyrus greggi* and two samples of the Columbia duskysnail (Table 3).

**Figure 4.**
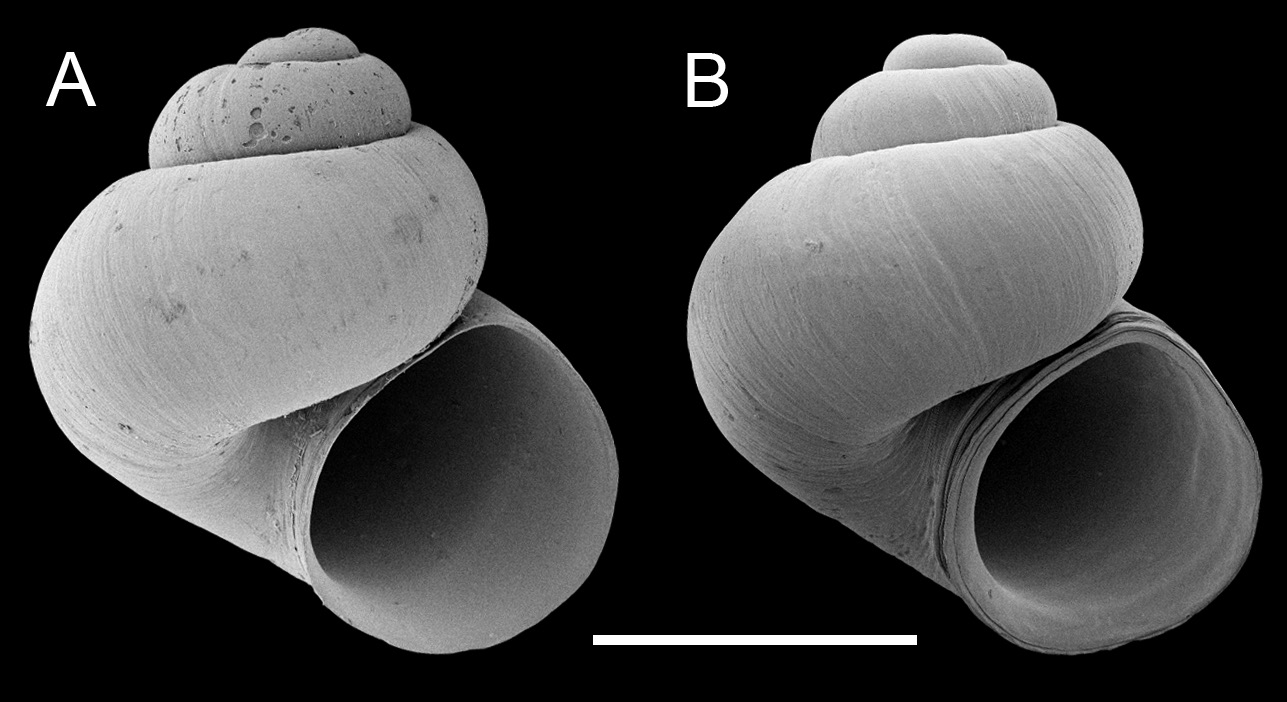
Scanning electron micrographs of shells of the Columbia duskysnail (**A** USNM 1256484) and *Colligyrus greggi* (**B** USNM 905375) having similarly sized spires. Scale bar: 1.0 mm.

Shell parameters (shell height, spire size and shape) and ANOVA results are reported in Table 3. Spire size overlapped considerably among specimens of the Columbia duskysnail and *Colligyrus greggi* (Figs 3–4) and was significantly associated with shell height (Pearson correlation, r^2^ = 0.73, *P* <0.01). The same patterns were observed for spire shape. Sample heterogeneity was highly significant for shell size, spire size, and spire shape (Table 3). *Colligyrus greggi* had significantly larger and more elongate spires (*P*<0.05) than the Columbia duskysnail in five of 10, and seven of 10 pairwise comparisons (among samples), respectively. However, the differences in these parameters were not significant in seven of eight comparisons between samples of the Columbia duskysnail and similar sized *Colligyrus greggi* (USNM 905275, USNM 1003672). The discriminant function analysis of the standard shell parameters delineated significant differences between the Columbia duskysnail and *Colligyrus greggi* (Wilk’s lambda = 0.5678, *F* = 9.0257, df = 7, *P* < 0.0001). However, the classification matrix correctly distinguished only 14/20 (70%) of the Columbia duskysnails while 67/71 (94%) of the *Colligyrus greggi* specimens were correctly distinguished. (This dataset is available from the first author on request.)

**Table 3.** Variation in shell parameters among samples of *Colligyrus greggi* and the Columbia duskysnail. Values are mean ± standard deviation, and range.

**Sample**	**SH (mm)**	**Spire size (SH-AH/AH)**	**Spire shape (SH-AH/WBW)**
*Colligyrus greggi*			
USNM 883531 (*N*=12)	2.65 ± 0.17 2.40–2.98	1.27 ± 0.09 1.11–1.42	0.88 ± 0.06 0.77–0.96
USNM 905375 (*N*=11)	2.14 ± 0.15 1.88–2.44	0.87 ± 0.11 0.72–1.10	0.65 ± 0.06 0.58–0.76
USNM 905382 (*N*=20)	2.64 ± 0.13 2.40–2.95	1.08 ± 0.07 1.00–1.23	0.78 ± 0.04 0.73–0.88
USNM 1003672 (*N*=11)	2.35 ± 0.07 2.25–2.47	1.03 ± 0.06 0.88–1.10	0.72 ± 0.04 0.64–0.77
USNM 1075739 (*N*=17)	2.82 ± 0.19 2.43–3.13	1.14 ± 0.10 0.99–1.34	0.83 ± 0.05 0.75–0.90
Columbia duskysnail			
USNM 1256484 (*N*=10)	2.34 ± 0.18 2.08–2.57	0.98 ± 0.08 0.64–0.80	0.72 ± 0.05 0.64–0.80
USNM 1256489 (*N*=10)	1.94 ± 0.26 1.67–2.47	0.95 ± 0.13 0.75–1.16	0.67 ± 0.06 0.57–0.78
*ANOVA	**F=43.717	**F=24.601	**F=35.301

* *DF* for all parameters was 6, 84

** Highly significant (*P*<0.01)

**Figure 5.**
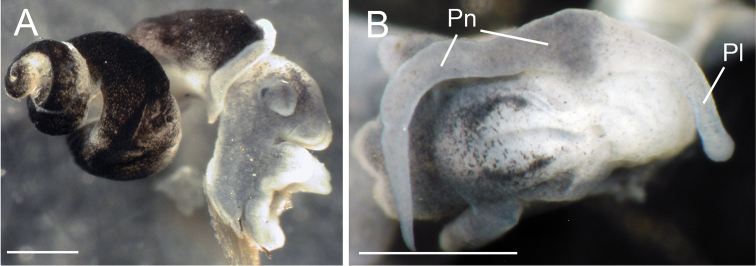
Photographs of ethanol preserved specimens of the Columbia duskysnail (USNM 1256484) showing pigmentation of body (**A**) and penis (**B**). Scale bars: 500 µm. **Pl** penial lobe, **Pn** penis.

**Figure 6.**
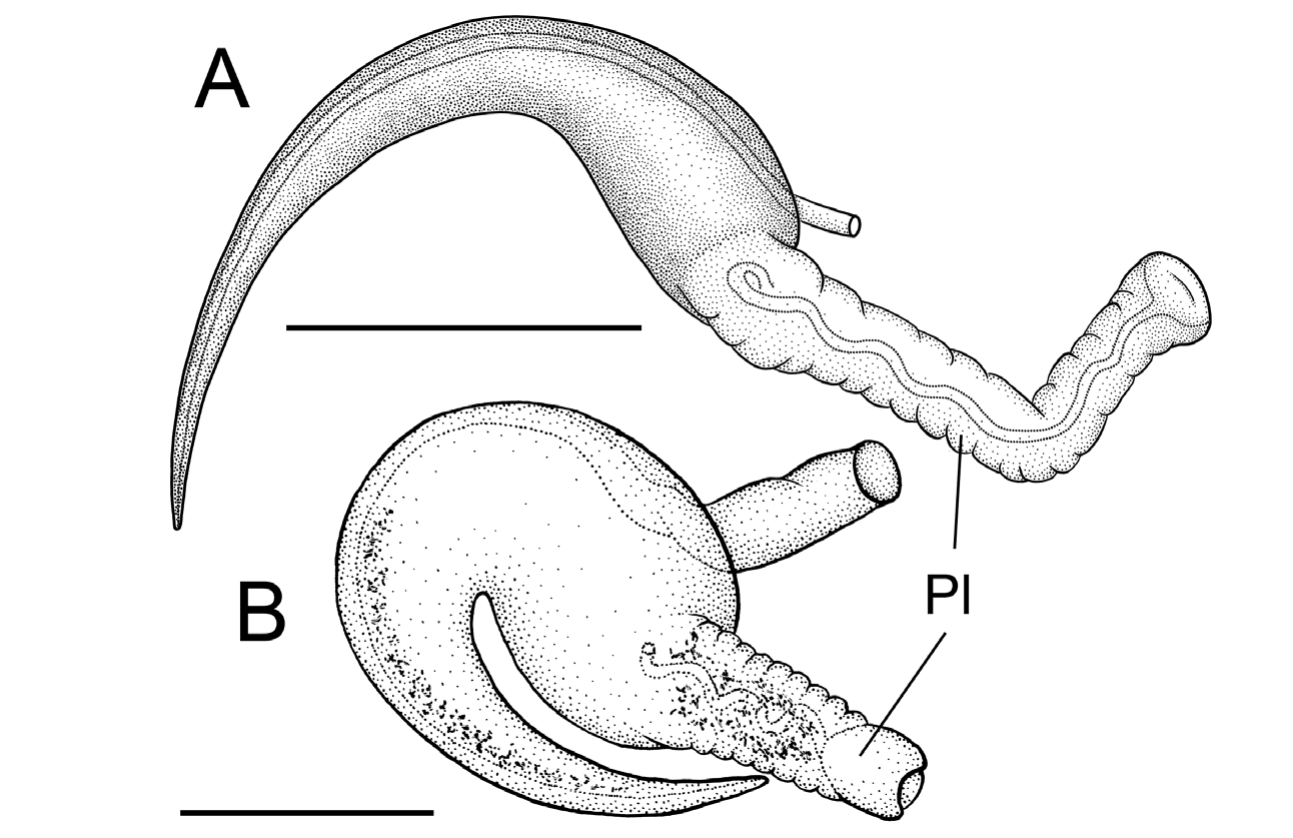
Dorsal views of penes of the Columbia duskysnail (**A** USNM 1256484) and *Colligyrus greggi* (**B** USNM 883531, reproduced from Hershler 1999, Fig. 2C). Scale bars: 250 µm. **Pl** penial lobe.

We were unable to confirm the purported differences in soft part pigmentation between the Columbia duskysnail and *Colligyrus greggi*. The pallial roof and visceral coil of the Columbia duskysnail is darkly pigmented and often black (Fig. 5A) as was described for *Colligyrus greggi* (Hershler 1999). The penis also conformed to that of *Colligyrus greggi* in having a basally concentrated internal core of dark pigment (Fig. 5B). The Columbia duskysnail closely resembled *Colligyrus greggi* in most other details (i.e., the number of cusps on the radular teeth, Table 4), although it appears to have a relatively longer penial lobe based on examination of a half dozen males (Fig. 6A–B).

**Table 4.** Radular cusp counts for *Colligyrus greggi* (from Hershler 1999) and the Columbia duskysnail (USNM 1256484).

	*Colligyrus greggi*	Columbia duskysnail
Central teeth, lateral cusps	4–7	4–7
Central teeth, basal cusps	1–2	1–2
Lateral teeth, cusps on inner side	2–4	3–5
Lateral teeth, cusps on outer side	3–4	4–5
Inner marginal teeth	24–27	24–30
Outer marginal teeth	25–33	23–29

## Discussion

Our molecular analysis further confirms a close relationship between the Columbia duskysnail and *Colligyrus greggi* and also indicates that populations of the former do not form an evolutionarily distinct, monophyletic unit. The COI sequence divergence between the Columbia duskysnail and *Colligyrus greggi* (2.1 ± 0.5%) is somewhat larger than differentiation within the latter (1.0 ± 0.3%) but considerably less than that among the three currently recognized congeners (8.7–12.1%). We also found that the Columbia duskysnail closely resembles *Colligyrus greggi* morphologically and cannot be consistently distinguished from it based on the diagnostic characters reported in grey literature (or other shell parameters). Consequently we conclude that the Columbia duskysnail is conspecific with *Colligyrus greggi*.

*Colligyrus greggi* can be added to a long list of plant and animal species that have broadly disjunct, coastal-inland distributions in the Pacific Northwest (Brunsfeld et al. 2001, Bjork 2010, Shafer et al. 2010). The full extent of the geographic range *Colligyrus greggi* is uncertain pending resolution of the taxonomic status of duskysnail populations in western Montana and northern Idaho. The *Colligyrus greggi* populations in the Mount Hood area are geographically isolated and somewhat differentiated genetically relative to other members of this species. There is also evidence of minor morphological differentiation of these animals—i.e., they tend to be smaller and have a longer penial lobe than other *Colligyrus greggi*. Collectively this evidence suggests that the populations in the Mount Hood area should be recognized as a distinct conservation unit (within *Colligyrus greggi*) that may merit monitoring and other protective measures.

As mentioned in the introduction to this paper, there are numerous taxonomically unstudied populations in the northwestern United States that may be assignable to *Colligyrus*; it is likely that some of these are new species. Although our findings have shown that the Columbia duskysnail cannot be considered a distinct congener, the undescribed populations in the Klamath Lake basin (KL) and Puget Sound area (COL7) merit further study as candidate species given that they differ from other *Colligyrus* lineages by 4.0–13.2% COI sequence divergence. The positioning of the latter outside of the *Colligyrus* clade, together with the unusual (near planispiral) shells of these snails suggests that they may belong to a previously unrecognized component of the North American amnicolid radiation.

### Material examined (* voucher material for new sequences presented herein)

*Colligyrus greggi*.—IDAHO. Bear Lake County. USNM 905382, spring at Saint Charles campground, Bear Lake basin, 42.11°N, 111.4662°W. USNM 905375, spring at Porcupine campground, Bear Lake basin, 42.0951°N, 111.5179°W. Fremont County. USNM 1003672, Otter Springs, upper Snake River basin, 44.1545°N, 111.2132°W. OREGON. Clackamas County. USNM 1019124, Oak Grove Fork, 0.24 km upstream from Timothy Lake, Willamette River basin, 45.11076°N, 121.76156°W. Hood River County. *USNM 1256484, small spring, Brooks Meadow, middle Columbia River basin, 45.41389°N, 121.52659°W. *USNM 1256483, spring tributary, Tony Creek, middle Columbia River basin, 45.49263°N, 121.70352°W. *USNM 1256485, Bottle Prairie, west of Eightmile Creek, middle Columbia River basin, 45.39471°N, 121.49992°W. *USNM 1256488, Bear Creek, side channel, Hood River Co., OR, 45.49386°N, 121.64251°W. Wasco County. *USNM 1256486, spring tributary, Ramsey Creek, middle Columbia River basin, 45.40065°N, 121.46345°W. *USNM 1256487, spring tributary, Clear Creek, Deschutes River basin, 45.14148°N, 121.58495°W. WYOMING. Sublette County. USNM 883531, Cliff Creek, tributary springs, upper Snake River basin, 43.2454°N, 110.5002°W.

*Colligyrus* sp.—WASHINGTON. Thurston County. *USNM 1258917, Allison Springs, Puget Sound drainage, 47.04432°N, 122.98454°W.
